# Age at Menarche, Cardiometabolic Disease Prevalence, and Disease Control in Korean Women: Analysis of the Korea National Health and Nutrition Examination Survey

**DOI:** 10.7150/ijms.137086

**Published:** 2026-07-30

**Authors:** Yuki Gen, Gyu-Na Lee, Kyung-Do Han, Yun Sung Jo

**Affiliations:** 1 Department of Obstetrics and Gynecology, St. Vincent's Hospital, College of Medicine, The Catholic University of Korea, Seoul, Republic of Korea.; 2 Department of Biomedicine & Health Science, The Catholic University of Korea, Seoul, Republic of Korea.; 3 Department of Statistics and Actuarial Science, Soongsil University, Seoul, Republic of Korea.

**Keywords:** age at menarche, menopause, type 2 diabetes mellitus, dyslipidemia, disease control, KNHANES

## Abstract

**Background:**

Early menarche is linked to adverse cardiometabolic outcomes, but its relationship with disease management remains unclear. We examined associations between menarcheal age and the prevalence and control of cardiometabolic diseases in Korean women.

**Methods:**

In 25,227 women aged ≥19 years from the Korea National Health and Nutrition Examination Survey (KNHANES), 2014-2023, menarcheal age was categorized as early (<12 years), intermediate (12-15 years; reference), and late (≥16 years). Multivariable logistic regression assessed the prevalence and control of type 2 diabetes mellitus (T2DM), hypercholesterolemia, and hyper-low-density lipoprotein (LDL) cholesterolemia, stratified by menopausal status.

**Results:**

Among premenopausal women, unadjusted comparisons favored the early-menarche group, reflecting age confounding; no significant association persisted after full adjustment for body mass index, lifestyle, and socioeconomic factors. Among postmenopausal women, late menarche was independently associated with lower odds of having hypercholesterolemia (OR = 0.809, 95% CI: 0.736-0.889; *p* < 0.001) and hyper-LDL cholesterolemia (OR = 0.815, 95% CI: 0.741-0.897; *p* < 0.001); no association with T2DM was observed. In contrast, among postmenopausal women who already had hyper-LDL cholesterolemia, those with late menarche were less likely to reach adequate LDL control than those with menarche at 12 to 15 years (OR = 0.814, 95% CI 0.693-0.956; *p* = 0.019). Although both odds ratios are below 1, they carry opposite clinical meaning, reflecting a lower prevalence of disease but poorer control once disease has developed.

**Conclusions:**

The cardiometabolic effects of menarcheal age appear life-stage dependent, extending beyond prevalence to disease management. In postmenopausal women, late menarche was associated with lower odds of dyslipidemia yet, paradoxically, with poorer LDL control once disease had developed, indicating that a lower observed risk should not justify less vigilant management. These findings support integrating menarcheal age into cardiometabolic risk assessment.

## Introduction

Menarche, the onset of menstruation, marks a critical physiological milestone in female development and serves as an objective, reproducible marker of pubertal timing along the female life course. Early menarche, typically defined as the onset of menstruation before the age of 12 years, has garnered increasing attention in epidemiological studies. Numerous studies have reported that early menarche is closely associated with an elevated risk of metabolic disorders in adulthood, including obesity, insulin resistance, and cardiovascular disease 1,2,3,4,5.

In Korea, the average age at menarche has shown a consistent downward trend over recent decades, a shift that has occurred alongside the rising prevalence of childhood obesity 6. For example, women born before 1935 experienced menarche at an average age of approximately 16.9 years, whereas this age dropped to approximately 12.5 years for those born between 2000 and 2004, with a more significant decrease observed in those with obesity 7. These secular shifts carry implications beyond reproductive biology, with growing evidence that earlier pubertal timing may increase the long-term burden of cardiometabolic disease and potentially affect how well those diseases are managed in later life.

However, studies simultaneously examining the relationship between age at menarche and disease prevalence and control remain scarce. For cardiometabolic diseases, disease control encompasses more than mere prevalence; it reflects a range of behavioral, socioeconomic, and clinical factors, including individual health behaviors, access to healthcare, medication adherence, and comorbidities. Thus, investigating whether age at menarche, as an early-life physiological indicator, is associated with the control of chronic diseases in adulthood has important implications from a health equity perspective.

Therefore, this study aimed to comprehensively evaluate the association between age at menarche and metabolic health outcomes in Korean women using nationally representative KNHANES data. Specifically, we examined whether age at menarche is associated with the prevalence of type 2 diabetes mellitus (T2DM), hypercholesterolemia, and hyper-LDL cholesterolemia, and further investigated whether menarcheal timing relates to indicators of disease management, including glycemic and lipid control, among individuals already diagnosed with these conditions.

## Methods

### Data Source and Study Population

The KNHANES is a nationally representative, population-based cross-sectional survey conducted by the Korea Disease Control and Prevention Agency (KDCA) since 1998. It is designed to assess the health status, health behaviors, and nutritional intake of the non-institutionalized Korean population. The survey employs a complex, multistage, stratified cluster sampling method and consists of three main components: a health interview, a health examination, and a nutrition survey 8. The KNHANES is conducted under the approval of the Institutional Review Board (IRB) of the KDCA and is regarded as a nationally approved public health survey 9. The current secondary analysis of de-identified public-access data was reviewed and approved by the IRB of The Catholic University of Korea (IRB No. VC25ZASI0323), with the requirement for informed consent waived.

This study utilized KNHANES data collected from 2014 to 2023. Initially, 40,876 female participants were identified, among whom 34,272 were aged 19 years or older. Of these, 9,045 women were excluded due to missing data on menopausal status-related factors (n = 6,281), reproductive factors (n = 503), chronic disease-related variables (n = 1,739), or covariates (n = 522). Ultimately, 25,227 women were included in the final analysis. The cohort comprised premenopausal women and women with natural menopause only; women with surgical or other non-natural menopause were excluded during cohort construction, so the postmenopausal stratum reflects natural menopause exclusively.

### Measurements and Lifestyle Habits

Baseline characteristics were obtained using standardized KNHANES questionnaires, including information on demographics, socioeconomic status, lifestyle behaviors, and clinical factors 9. Age at menarche, which was self-reported by participants, was categorized into early (< 12 years), intermediate (12-15 years), and late (≥ 16 years), with the intermediate group used as the reference category. These thresholds follow established convention: a cutoff of < 12 years is the most widely used definition of early menarche and the threshold most consistently associated with adverse cardiometabolic outcomes 1,2; the 12-15-year band represents the normative range encompassing the contemporary Korean mean age at menarche (approximately 12.5-13 years) 6,7; and ≥ 16 years denotes late menarche, consistent with prior KNHANES-based studies of menarcheal timing in Korean women 5. Income was categorized into quartiles, with the lowest quartile defined as the low-income group 10. Education level was classified as high (≥ 13 years of education) or low (< 13 years). Employment status was recorded as employed or unemployed based on current economic activity. Smoking status was assessed as ever smoking, defined as a self-reported lifetime cumulative consumption of ≥ 100 cigarettes. Alcohol intake was calculated by multiplying the average drinking frequency (days per month) by the typical amount consumed per occasion (in milliliters), adjusted for the type of alcoholic beverage, and converted into grams of pure alcohol consumed per day. Participants with an average alcohol consumption exceeding 30 grams per day were classified as heavy drinkers 11. BMI was calculated as body weight in kilograms divided by the square of height in meters. Obesity was defined as a BMI ≥ 25 kg/m² according to the Asia-Pacific criteria of the World Health Organization guidelines 12. Abdominal obesity was defined as a waist circumference ≥ 85 cm in women, in accordance with the criteria established by the Korean Society for the Study of Obesity 13. Participants were classified as physically active if they met any of the following criteria: (i) ≥ 150 minutes/week of moderate-intensity activity, (ii) ≥ 75 minutes/week of vigorous-intensity activity, (iii) an equivalent combination of both, or (iv) strength training ≥ 1 time/week 11.

### Anthropometric and Clinical Measurements

Anthropometric measurements including height, weight, BMI, waist circumference, systolic and diastolic blood pressure, and fasting plasma glucose were obtained by trained medical personnel during standardized health examinations. Biochemical markers including HbA1c, total cholesterol, high-density lipoprotein (HDL) cholesterol, LDL cholesterol, and triglycerides were measured from fasting blood samples. Triglyceride levels were reported as geometric means with 95% confidence intervals due to their right-skewed distribution.

### Definitions of Cardiometabolic Diseases and Control

T2DM was defined as a fasting plasma glucose level ≥ 126 mg/dL 14, a glycated hemoglobin A1c (HbA1c) level ≥ 6.5%, current use of oral hypoglycemic agents or insulin, or a self-reported physician diagnosis of diabetes. Among participants with T2DM, adequate glycemic control was defined as a HbA1c level < 6.5% 15.

Hypercholesterolemia was defined as a fasting total cholesterol level ≥ 240 mg/dL or current use of lipid-lowering medication. Control of hypercholesterolemia was defined as a fasting total cholesterol level < 200 mg/dL 16.

Hyper-LDL cholesterolemia was defined as a fasting LDL cholesterol level ≥ 160 mg/dL or current use of lipid-lowering medication. Control of hyper-LDL cholesterolemia was defined as a fasting LDL cholesterol level < 130 mg/dL 16.

### Statistical Analysis

All analyses accounted for the KNHANES complex survey design, incorporating sampling weights, stratification, and clustering to produce nationally representative estimates; sampling weights were rescaled in accordance with the analytic guidelines of the Korea Disease Control and Prevention Agency to account for the combined 10-year (2014-2023) survey period. Baseline characteristics were summarized as weighted means ± SE for continuous variables and as weighted percentages with SE for categorical variables; geometric means with 95% confidence intervals (CIs) were reported for non-normally distributed variables such as triglycerides. For Table 1, between-group comparisons used design-based methods: survey-weighted linear regression (PROC SURVEYREG) for continuous variables and the Rao-Scott design-adjusted chi-square test (PROC SURVEYFREQ) for categorical variables. Associations between age at menarche (< 12, 12-15 [reference], ≥ 16 years) and the prevalence and control of T2DM, hypercholesterolemia, and hyper-LDL cholesterolemia were assessed using survey-weighted multivariable logistic regression (PROC SURVEYLOGISTIC), yielding adjusted odds ratios (ORs) with 95% CIs, with separate models for premenopausal and postmenopausal women. Three models were fitted sequentially: Model 1 was unadjusted; Model 2 was adjusted for age; and Model 3 was further adjusted for BMI, educational attainment (< 13 vs. ≥ 13 years), income level (lowest quartile vs. others), smoking status, alcohol intake, physical activity, and hypertension, as well as hypercholesterolemia (for diabetes models) or diabetes (for cholesterol models). Descriptive comparisons are presented dichotomously (< 12 vs. ≥ 12 years) to focus on the early-menarche threshold most frequently examined in prior literature, while regression analyses use the three-category classification (< 12, 12-15, ≥ 16 years) to resolve the late-menarche group separately. To allow direct comparison under a common grouping, prevalence and disease-control results are additionally reported under the three-category classification (Tables 3-4) and the dichotomous classification (Supplementary Tables S1-S2). All statistical analyses were performed using SAS version 9.4 (SAS Institute, Inc., Cary, NC, USA), and two-sided *P* values < 0.05 were considered statistically significant. The study was conducted and reported in accordance with the STROBE Statement for cross-sectional studies.

## Results

### Baseline Characteristics of Participants by Age at Menarche

Table 1 presents baseline characteristics of 12,753 premenopausal and 12,474 postmenopausal women, stratified by age at menarche (< 12 vs. ≥ 12 years) within menopausal strata. Among premenopausal women, early menarche (< 12 years) was associated with a substantially younger mean age (30.96 vs. 37.11 years; *p* < 0.001), reflecting Korea's well-documented secular decline in menarcheal age. Concordant with this age difference, the early-menarche group showed significantly lower crude blood pressure, total cholesterol, fasting glucose, HbA1c, and triglyceride levels; however, these differences should be interpreted with caution given the marked age imbalance between groups. Despite their younger age, the early-menarche group exhibited a significantly higher BMI (23.33 vs. 22.57 kg/m²; *p* < 0.001), and a higher prevalence of obesity (27.53% vs. 21.04%; *p* < 0.001) and abdominal obesity (18.54% vs. 16.13%; *p* = 0.010), consistent with the well-established association between childhood adiposity and earlier puberty. They also had higher rates of meeting recommended physical activity criteria (56.95% vs. 49.93%; *p* < 0.001) and higher educational attainment (61.13% vs. 57.31% with ≥ 13 years; *p* = 0.004).

In postmenopausal women, the early-menarche group was again significantly younger (58.99 vs. 63.59 years; *p* < 0.001) and showed lower systolic blood pressure (119.82 vs. 123.59 mmHg; *p* = 0.003) and higher HDL-cholesterol levels (57.39 vs. 54.72 mg/dL; *p* = 0.010). Additionally, postmenopausal women with early menarche had a significantly lower prevalence of low income (19.43% vs. 27.18%; *p* = 0.012) and higher rates of higher educational attainment (31.26% vs. 15.35%; *p* < 0.001) compared to those with menarche at ≥ 12 years, a pattern again consistent with secular cohort effects. Body weight was also significantly greater in the early-menarche group (59.25 vs. 57.78 kg; *p* = 0.034), while other cardiometabolic indicators did not reach statistical significance.

### Prevalence of Cardiometabolic Diseases by Age at Menarche

Table 2 presents the crude weighted prevalence of T2DM, hypercholesterolemia, and hyper-LDL cholesterolemia by menarche category (< 12 vs. ≥ 12 years) and menopausal status. Among premenopausal women, those with early menarche had a significantly lower crude prevalence of hypercholesterolemia (7.25% vs. 9.10%, *p* = 0.014) and hyper-LDL cholesterolemia (6.66% vs. 8.13%, *p* = 0.044) compared to the ≥ 12 group. The crude prevalence of T2DM did not differ significantly between groups (2.45% vs. 2.95%, *p* = 0.216). Among postmenopausal women, no statistically significant differences in the crude prevalence of any condition were observed. Whether these crude differences in dyslipidemia prevalence among premenopausal women persist after adjustment for sociodemographic and lifestyle confounders was examined using multivariable logistic regression with the full three-category classification (< 12, 12-15, and ≥ 16 years), as presented in Table 3.

In the fully adjusted model (Model 3), age at menarche was not significantly associated with the prevalence of any of the three cardiometabolic conditions among premenopausal women, indicating complete attenuation of the crude associations observed in Table 2. The estimates for the premenopausal late-menarche (≥ 16 years) subgroup, particularly for type 2 diabetes, were based on a small number of cases and should therefore be interpreted with caution, as reflected in their wide confidence intervals and instability across models (subgroup sizes and case counts are reported in the Table 3 footnote).

Among postmenopausal women, late menarche (≥ 16 years) was significantly and independently associated with a lower prevalence of dyslipidemia. Compared to women with menarche at 12-15 years, those with menarche at ≥ 16 years had substantially lower odds of both hypercholesterolemia (OR = 0.809, 95% CI: 0.736-0.889; *p* < 0.001) and hyper-LDL cholesterolemia (OR = 0.815, 95% CI: 0.741-0.897; *p* < 0.001) in the fully adjusted model, corresponding to approximately a 19% lower odds for each outcome compared with women whose menarche occurred at 12-15 years. No significant association was observed between late menarche and T2DM prevalence in this group.

Early menarche (< 12 years) was not significantly associated with the prevalence of T2DM, hypercholesterolemia, or hyper-LDL cholesterolemia in either premenopausal or postmenopausal women compared to the reference group (12-15 years).

### Control of Cardiometabolic Diseases by Age at Menarche

Table 4 presents multivariable-adjusted odds ratios for disease control among women with established cardiometabolic conditions, stratified by menopausal status. Among premenopausal women, age at menarche was not independently associated with control of any cardiometabolic condition in the fully adjusted model. The transient association observed in Model 2, in which the early-menarche group showed higher odds of adequate cholesterol and LDL control after age adjustment alone, was fully explained by BMI, lifestyle, and socioeconomic covariates. Glycemic control analyses for T2DM should be interpreted cautiously, as the low premenopausal T2DM prevalence of approximately 2.95% yielded a small affected subgroup with limited statistical power.

Among postmenopausal women, late menarche was independently associated with lower odds of achieving adequate LDL cholesterol control compared with women whose menarche occurred at 12-15 years in the fully adjusted model (OR = 0.814, 95% CI: 0.693-0.956; *p* = 0.019). This finding contrasts with the lower hyper-LDL prevalence observed in the same late-menarche group, as shown in Table 3, and this dissociation is discussed below in the Discussion. By contrast, early menarche showed no significant association with the control of any cardiometabolic condition, and the control of T2DM and hypercholesterolemia in postmenopausal women did not differ significantly across menarche groups.

## Discussion

This nationally representative, decade-spanning study of Korean women examined the relationship between age at menarche and cardiometabolic health across the life course, stratifying all analyses by menopausal status to account for the profound hormonal transition that characterizes midlife in women. Four principal findings emerged. First, in premenopausal women the unadjusted descriptive profile appeared to favor the early-menarche group across multiple cardiometabolic markers, including blood pressure, fasting glucose, HbA1c, total cholesterol, and triglyceride levels, but this apparent advantage likely reflected age confounding, as the early-menarche group was substantially younger than the later-menarche group, consistent with Korea's pronounced secular decline in menarcheal age. Once age was accounted for (Model 2), the direction reversed, and early menarche was significantly associated with higher odds of all three cardiometabolic conditions, indicating that chronological age had masked a genuine adverse signal. Second, this age-revealed association was fully attenuated after additional adjustment for BMI, lifestyle, and socioeconomic factors (Model 3), indicating that the excess risk associated with early menarche is largely mediated by modifiable behavioral and environmental factors rather than by menarcheal timing itself. Third, late menarche (≥ 16 years) was independently associated with a lower prevalence of dyslipidemia among postmenopausal women. Fourth, and most notably, late menarche was paradoxically associated with lower odds of achieving adequate LDL cholesterol control among postmenopausal women with established hyper-LDL cholesterolemia, a finding with direct clinical implications. Together, these results suggest that the cardiometabolic consequences of menarcheal timing operate through distinct pathways at different life stages and manifest differently across disease prevalence and disease control outcomes.

The age-revealed association between early menarche and adverse cardiometabolic outcomes in premenopausal women is directionally consistent with a substantial body of prior evidence 1,2,3,4,5. In a cohort study using earlier KNHANES data, early menarche was identified as a significant predictor of metabolic syndrome and insulin resistance among premenopausal Korean women 1, and a systematic review of 16 studies confirmed similar associations across diverse populations 2.

The biological substrate linking early menarche to adult cardiometabolic disease operates through two interconnected pathways. First, childhood adiposity serves as a shared upstream driver: excess adipose tissue promotes early secretion of leptin and insulin-like growth factor-1, which together trigger premature activation of the hypothalamic-pituitary-ovarian axis 17 while concurrently establishing a pro-inflammatory, insulin-resistant metabolic milieu that may persist into adulthood 18. Second, the resulting earlier menarche prolongs lifetime exposure to ovarian estrogens, which stimulates hepatic triglyceride synthesis, enhances very-low-density lipoprotein (VLDL) secretion 19, and promotes redistribution of body fat toward visceral adipose depots 17,20, each independently contributing to long-term cardiometabolic risk.

In our cohort, this biological signal was almost entirely masked in the unadjusted comparison (Model 1) because women with early menarche were substantially younger than those with later menarche (30.96 vs. 37.11 years), reflecting Korea's pronounced secular decline in menarcheal age 7. This age imbalance suppressed the crude odds ratios below unity, producing apparent inverse associations that reflected age confounding rather than a true protective effect of early menarche. A similar phenomenon was observed in a large prospective study of 300,000 Chinese women, in which secular birth-cohort effects were shown to systematically confound cross-sectional analyses of menarcheal age 21. Once age was accounted for (Model 2), the odds ratios reversed above unity for all three outcomes, recovering an association consistent with the mechanistic pathways outlined above.

By contrast, no significant association between menarcheal age and T2DM prevalence was observed in any model. This null finding likely reflects the small number of T2DM cases among premenopausal women (~2.95%) and the broader case definition used in KNHANES, and differs somewhat from prior meta-analytic evidence 22 suggesting a modest BMI-mediated association detectable mainly in longitudinal designs.

In postmenopausal women, late menarche was independently associated with lower odds of hypercholesterolemia (OR = 0.809, 95% CI: 0.736-0.889; *p* < 0.001) and hyper-LDL cholesterolemia (OR = 0.815, 95% CI: 0.741-0.897; *p* < 0.001). This effect emerging only after menopause likely reflects an estrogen-unmasking phenomenon: during the reproductive years, endogenous estrogen maintains a favorable lipid profile 23 and may obscure the cumulative metabolic advantage of women with shorter lifetime estrogen exposure. Once estrogen declines at menopause, this advantage becomes clinically detectable. This interpretation is supported by longitudinal SWAN data showing that LDL cholesterol and apolipoprotein B rise sharply across the menopausal transition independent of aging 24,25, and by Mendelian randomization evidence linking older menarche with a less atherogenic lipid profile 26. A meta-analysis pooling multiple cohorts has further linked older menarche with lower long-term cardiovascular mortality, providing outcome-level support 27.

A clinically notable finding is the paradoxical association between late menarche and poorer LDL control among postmenopausal women with established disease (OR = 0.814, 95% CI: 0.693-0.956; *p* = 0.019). Three non-mutually exclusive explanations may apply. First, women with late menarche may be subject to less vigilant screening and less intensive lipid-lowering therapy because of their lower a priori risk, consistent with the well-documented undertreatment of dyslipidemia in women perceived to be at low cardiovascular risk 16,28. Second, dyslipidemia developing in this biologically protected group may represent a qualitatively distinct form that is less responsive to standard first-line therapy, reflecting sex-specific differences in statin metabolism, although direct evidence remains limited 23,28. Third, lower perceived risk may reduce disease awareness and treatment adherence, a behavioral gap particularly relevant among Korean women, whose dyslipidemia treatment goal attainment remains suboptimal 16. These hypotheses warrant prospective testing using objective adherence data and detailed lipid profiling.

Among premenopausal women, no independent association between menarcheal age and disease control was observed in the fully adjusted model; this likely reflects the shorter disease duration and limited statistical power available within this stratum.

From a health equity perspective, our cohort showed an unusual pattern: early-menarche women had higher education and physical activity rather than the socioeconomic disadvantage typically reported elsewhere. This likely reflects the dominance of secular cohort effects, since earlier-menarche women tended to belong to younger birth cohorts that benefited from broader improvements in education and health behaviors in Korea. Globally, however, early menarche remains disproportionately prevalent in disadvantaged populations 3, and the compounding of biological vulnerability with structural barriers to care may contribute to cardiometabolic disparities.

The principal strengths of this study include the use of a nationally representative dataset spanning 10 years of KNHANES cycles 8,9, simultaneous evaluation of disease prevalence and control, and consistent stratification by menopausal status. Several limitations should be acknowledged. Because age at menarche and the cardiometabolic outcomes were ascertained at a single point in time, the temporal ordering between menarcheal timing and the measured outcomes cannot be established, and all associations reported here should be interpreted as cross-sectional rather than causal. Age at menarche was self-reported and subject to potential recall bias, although recalled menarcheal age generally shows reasonable agreement with prospectively recorded values. Statin use, medication adherence, and detailed lipid profiles were unavailable, which limits interpretation of the LDL control paradox 28; relatedly, because hypercholesterolemia and hyper-LDL cholesterolemia were defined in part by current lipid-lowering medication use, we could not perform a sensitivity analysis restricted to untreated, biochemically defined disease, as excluding treated participants would alter the case definition and remove the individuals most relevant to the disease-control analysis. Menopausal hormone therapy use and age at menopause were not included in our models; because exogenous estrogen affects lipid metabolism and time since menopause more directly reflects the duration of estrogen deficiency, their omission may have introduced residual confounding in postmenopausal women. In addition, age at menarche was modeled as a categorical rather than a continuous variable; although this facilitates clinical interpretation and comparison with prior literature, it does not capture potential dose-response relationships across the full range of menarcheal age. The relatively low T2DM prevalence among premenopausal women (~2.95%) and the small early-menarche subgroup among postmenopausal women (n = 244, 2.0%) limited statistical precision and increased susceptibility to type II error. Lastly, the absence of longitudinal body composition data precluded examination of adiposity trajectories as mediating pathways 17,20,29. Future prospective studies incorporating medication adherence data and life-course adiposity measures will be needed to clarify the causal mechanisms linking menarcheal timing to chronic disease control in women. Finally, although these nationally representative findings are concordant with prior cohort studies, meta-analyses, and Mendelian randomization data, external validation remains warranted. Replication in other national databases, prospective cohorts, or linked clinical registries, particularly longitudinal studies incorporating treatment data, would help establish the robustness and reproducibility of these associations.

## Conclusion

This nationally representative analysis demonstrates that the cardiometabolic consequences of menarcheal age are life-stage dependent and extend beyond disease prevalence to include disease control. In premenopausal women, the risk associated with early menarche appears to be largely mediated through modifiable factors such as adiposity, lifestyle, and socioeconomic conditions, suggesting that preventive interventions during adolescence and early adulthood may attenuate this risk. In postmenopausal women, late menarche was independently associated with a lower prevalence of dyslipidemia, yet, paradoxically, with poorer LDL cholesterol control among those with established disease. This finding underscores that a lower a priori risk should not be interpreted as a justification for less vigilant clinical management. Our findings support the integration of menarcheal age into cardiometabolic risk assessment across the female life course, with particular attention to lipid monitoring and treatment adherence in postmenopausal women regardless of pubertal timing. Future longitudinal studies incorporating medication adherence and detailed lipid profiling will be needed to clarify the mechanisms underlying this paradox.

## Supplementary Material

Supplementary tables.

## Figures and Tables

**Table 1:** Baseline Characteristics of Premenopausal and Postmenopausal Women by Age at Menarche (<12 vs. ≥12 Years)

	Premenopausal women		*p* -value	Postmenopausal women		*P* value
Age of menarche (years)	< 12 (n = 2,335)	≥ 12 (n = 10,418)		< 12 (n = 244)	≥ 12 (n = 12,230)	
Age, years	30.96 ± 0.20	37.11 ± 0.12	< 0.001	58.99 ± 0.53	63.59 ± 0.11	< 0.001
Height, cm	161.01 ± 0.12	160.99 ± 0.07	0.838	155.82 ± 0.35	155.02 ± 0.07	0.022
Weight, kg	60.53 ± 0.29	58.51 ± 0.12	< 0.001	59.25 ± 0.69	57.78 ± 0.09	0.034
BMI, kg/m²	23.33 ± 0.11	22.57 ± 0.05	< 0.001	24.37 ± 0.25	24.04 ± 0.04	0.195
Waist Circumference, cm	76.37 ± 0.26	75.77 ± 0.12	0.032	82.53 ± 0.66	82.43 ± 0.11	0.882
Obesity	27.53 (1.03)	21.04 (0.47)	< 0.001	38.13 (3.49)	34.75 (0.53)	0.329
Abdominal obesity	18.54 (0.88)	16.13 (0.44)	0.010	38.05 (3.53)	37.61 (0.54)	0.903
Ever smoking	15.34 (0.89)	13.02 (0.41)	0.011	7.78 (1.81)	6.14 (0.27)	0.319
Heavy drinking	9.01 (0.69)	8.14 (0.31)	0.217	3.85 (1.38)	2.59 (0.18)	0.271
Physical activity	56.95 (1.19)	49.93 (0.58)	< 0.001	37.39 (3.73)	37.88 (0.55)	0.896
Occupation	61.32 (1.15)	62.10 (0.59)	0.538	49.64 (3.63)	45.91 (0.58)	0.308
Low income	8.41 (0.71)	7.12 (0.35)	0.073	19.43 (2.74)	27.18 (0.55)	0.012
Education (≥13 years)	61.13 (1.20)	57.31 (0.65)	0.004	31.26 (3.57)	15.35 (0.47)	< 0.001
SBP, mmHg	107.23 ± 0.27	108.55 ± 0.15	< 0.001	119.82 ± 1.26	123.59 ± 0.20	0.003
DBP, mmHg	70.68 ± 0.22	71.71 ± 0.11	< 0.001	74.31 ± 0.73	74.51 ± 0.11	0.786
Fasting glucose, mg/dL	91.76 ± 0.32	93.07 ± 0.18	< 0.001	103.42 ± 1.97	102.62 ± 0.23	0.689
HbA1c, %	5.34 ± 0.01	5.40 ± 0.01	< 0.001	5.91 ± 0.06	5.90 ± 0.01	0.891
Total cholesterol, mg/dL	186.88 ± 0.85	189.03 ± 0.38	0.019	201.14 ± 3.07	196.44 ± 0.44	0.127
HDL cholesterol, mg/dL	59.07 ± 0.33	58.73 ± 0.16	0.321	57.39 ± 1.02	54.72 ± 0.16	0.010
LDL cholesterol, mg/dL	109.97 ± 0.80	111.63 ± 0.34	0.051	121.51 ± 2.88	117.71 ± 0.40	0.189
* Triglyceride, mg/dL	80.11 (78.35-81.92)	83.46 (82.51-84.42)	0.001	103.05 (96.81-109.69)	108.33 (107.14-109.53)	0.122

Data were presented as a weighted % (SE) or mean±SE, * Geometric mean (95% CI)

**Table 2:** Prevalence of Cardiometabolic Diseases by Age at Menarche (<12 vs. ≥12 Years) Among Premenopausal and Postmenopausal Women

Age of menarche (years)	Premenopausal women		*p* -value	Postmenopausal women		*P value*
	< 12	≥ 12		< 12	≥ 12	
	(n = 2,335)	(n = 10,418)		(n = 244)	(n = 12,230)	
Diabetes	2.45 (1.79 - 3.11)	2.95 (2.59 - 3.32)	0.216	19.12 (13.38 - 24.86)	18.34 (17.54 - 19.14)	0.787
Hypercholesterolemia	7.25 (6.03 - 8.47)	9.10 (8.48 - 9.73)	0.014	44.39 (36.91 - 51.88)	41.54 (40.50 - 42.58)	0.457
Hyper-LDL cholesterolemia	6.66 (5.46 - 7.87)	8.13 (7.54 - 8.72)	0.044	44.22 (36.72 - 51.71)	40.13 (39.09 - 41.16)	0.282

Data were presented as a weighted % (95% CI)

**Table 3:** Multivariable-Adjusted Odds Ratios (95% CI) for the Association Between Age at Menarche (<12, 12-15, ≥16 Years) and the Prevalence of Cardiometabolic Diseases, with Unweighted Subgroup Sizes and Event Counts, Stratified by Menopausal Status

Prevalence	Premenopausal women					Postmenopausal women				
	Unweighted, n		OR (95% CI)			Unweighted, n		OR (95% CI)		
	Total	Event	Model 1	Model 2	Model 3	Total	Event	Model 1	Model 2	Model 3
Type 2 diabetes mellitus										
< 12	2335	64	0.886 (0.649, 1.208)	**1.520** **(1.096, 2.109)**	1.002 (0.698, 1.439)	244	48	1.176 (0.808, 1.711)	1.352 (0.922, 1.981)	1.304 (0.903, 1.883)
12-15 (Ref.)	9731	294	1 (Ref.)	1 (Ref.)	1 (Ref.)	7573	1343	1 (Ref.)	1 (Ref.)	1 (Ref.)
≥ 16	687	41	**2.165** **(1.474, 3.180)**	**1.579** **(1.065, 2.342)**	1.442 (0.908, 2.289)	4657	1031	**1.348** **(1.214, 1.497)**	0.955 (0.853, 1.068)	0.946 (0.843, 1.063)
*P value*			*< 0.001*	*0.005*	*0.299*			*< 0.001*	*0.204*	*0.220*
Hypercholesterolemia										
< 12	2335	174	**0.805** **(0.660, 0.983)**	**1.315** **(1.071, 1.614)**	1.089 (0.878, 1.352)	244	105	1.096 (0.805, 1.493)	1.166 (0.854, 1.592)	1.111 (0.818, 1.509)
12-15 (Ref.)	9731	914	1 (Ref.)	1 (Ref.)	1 (Ref.)	7573	3237	1 (Ref.)	1 (Ref.)	1 (Ref.)
≥ 16	687	85	**1.508** **(1.165, 1.951)**	1.125 (0.863, 1.466)	1.095 (0.828, 1.449)	4657	1916	0.933 (0.855, 1.017)	**0.792** **(0.722, 0.868)**	**0.809** **(0.736, 0.889)**
*P value*			*< 0.001*	*0.027*	*0.622*			*0.212*	*< 0.001*	*< 0.001*
Hyper-LDL cholesterolemia										
< 12	2335	158	0.834 (0.675, 1.030)	**1.329** **(1.065, 1.659)**	1.083 (0.855, 1.372)	244	104	1.169 (0.860, 1.591)	1.258 (0.922, 1.718)	1.198 (0.882, 1.628)
12-15 (Ref.)	9731	815	1 (Ref.)	1 (Ref.)	1 (Ref.)	7573	3121	1 (Ref.)	1 (Ref.)	1 (Ref.)
≥ 16	687	77	**1.528** **(1.166, 2.002)**	1.155 (0.878, 1.520)	1.094 (0.814, 1.470)	4657	1884	0.968 (0.887, 1.056)	**0.800** **(0.729, 0.878)**	**0.815** **(0.741, 0.897)**
*P value*			*0.001*	*0.031*	*0.690*			*0.435*	*< 0.001*	*< 0.001*

Data are odds ratios (95% CI); reference category, 12-15 years. Total, unweighted participants per subgroup; Event, number with the disease. Bold, 95% CI excluding 1. P values are the overall (global) test of association for age at menarche across the three categories; significance of each group versus the 12-15-year reference is indicated by whether its 95% CI excludes 1. Model 1, unadjusted; Model 2, adjusted for age; Model 3, adjusted for age, BMI, education (<13 vs. ≥13 years), low income, ever smoking, heavy drinking, aerobic physical activity, and hypertension, plus hypercholesterolemia (diabetes models) or diabetes (cholesterol models).

**Table 4:** Multivariable-Adjusted Odds Ratios (95% CI) for the Association Between Age at Menarche (<12, 12-15, ≥16 Years) and the Control of Cardiometabolic Diseases, with Unweighted Subgroup Sizes and Event Counts, Stratified by Menopausal Status

Control	Premenopausal women					Postmenopausal women				
	Unweighted, n		OR (95% CI)			Unweighted, n		OR (95% CI)		
	Total	Event	Model 1	Model 2	Model 3	Total	Event	Model 1	Model 2	Model 3
Type 2 diabetes mellitus										
< 12	64	17	0.801 (0.415, 1.544)	0.669 (0.331, 1.352)	0.743 (0.342, 1.614)	48	12	0.833 (0.399, 1.737)	0.849 (0.407, 1.768)	0.804 (0.388, 1.664)
12-15 (Ref.)	294	78	1 (Ref.)	1 (Ref.)	1 (Ref.)	1343	349	1 (Ref.)	1 (Ref.)	1 (Ref.)
≥ 16	41	8	1.017 (0.400, 2.588)	1.104 (0.418, 2.916)	1.113 (0.399, 3.106)	1031	296	1.037 (0.838, 1.285)	0.999 (0.803, 1.243)	1.030 (0.823, 1.289)
*P value*			*0.795*	*0.491*	*0.715*			*0.825*	*0.908*	*0.802*
Hypercholesterolemia										
< 12	174	32	0.899 (0.569, 1.421)	**1.695** **(1.030, 2.788)**	1.296 (0.742, 2.262)	105	55	0.804 (0.521, 1.240)	0.934 (0.599, 1.454)	0.847 (0.533, 1.345)
12-15 (Ref.)	914	199	1 (Ref.)	1 (Ref.)	1 (Ref.)	3237	1837	1 (Ref.)	1 (Ref.)	1 (Ref.)
≥ 16	85	20	1.474 (0.807, 2.692)	1.273 (0.677, 2.393)	1.070 (0.487, 2.353)	1916	1216	**1.351** **(1.180, 1.546)**	0.896 (0.774, 1.036)	0.863 (0.740, 1.007)
*P value*			*0.377*	*0.090*	*0.650*			*< 0.001*	*0.324*	*0.151*
Hyper-LDL cholesterolemia										
< 12	158	36	0.977 (0.631, 1.512)	**1.876** **(1.168, 3.013)**	1.601 (0.946, 2.711)	104	60	0.687 (0.442, 1.068)	0.804 (0.516, 1.251)	0.707 (0.447, 1.119)
12-15 (Ref.)	815	211	1 (Ref.)	1 (Ref.)	1 (Ref.)	3121	1972	1 (Ref.)	1 (Ref.)	1 (Ref.)
≥ 16	77	21	1.397 (0.761, 2.564)	1.115 (0.588, 2.116)	1.085 (0.493, 2.388)	1884	1299	**1.269** **(1.102, 1.461)**	**0.842** **(0.724, 0.980)**	**0.814** **(0.693, 0.956)**
*P value*			*0.548*	*0.032*	*0.209*			*0.001*	*0.062*	*0.019*

Data are odds ratios (95% CI); reference category, 12-15 years. Total, unweighted participants per subgroup; Event, number achieving adequate control. Bold, 95% CI excluding 1. P values are the overall (global) test of association for age at menarche across the three categories; significance of each group versus the 12-15-year reference is indicated by whether its 95% CI excludes 1. Model 1, unadjusted; Model 2, adjusted for age; Model 3, adjusted for age, BMI, education (<13 vs. ≥13 years), low income, ever smoking, heavy drinking, aerobic physical activity, and hypertension, plus hypercholesterolemia (diabetes models) or diabetes (cholesterol models).

## Data Availability

The datasets analyzed in the current study are publicly available from the Korea National Health and Nutrition Examination Survey (KNHANES) website operated by the Korea Disease Control and Prevention Agency (https://knhanes.kdca.go.kr) upon completion of the standard data-user registration process. Statistical analysis code used in this study is available from the corresponding author upon reasonable request.

## Abbreviations

KNHANES: Korea National Health and Nutrition Examination Survey
T2DM: Type 2 diabetes mellitus
LDL: Low-density lipoprotein
KDCA: Korea Disease Control and Prevention Agency
IRB: Institutional Review Board
BMI: Body mass index
SE: Standard error
HDL: High-density lipoprotein
HbA1c: Glycated Hemoglobin A1c
OR: Odds ratio
CI: Confidence interval
SBP: Systolic blood pressure
DBP: Diastolic blood pressure
TG: Triglyceride
VLDL: Very-low-density lipoprotein
