# Supplementary material for: Age at Menarche, Cardiometabolic Disease Prevalence, and Disease Control in Korean Women: Analysis of the Korea National Health and Nutrition Examination Survey
Source: Int J Med Sci. 2026 Jul 30;23(9):2911–20. doi: 10.7150/ijms.137086 (PMC13539909; doi:10.7150/ijms.137086)
Supplement: Supplementary file 1 [file ijmsv23p2911s1.pdf]

Supplementary Table S1. Cardiometabolic disease prevalence by age at menarche under the dichotomous classification (<12 vs. ≥12 years), with unweighted subgroup sizes and event counts, stratified by menopausal status.

**Prevalence**	**Premenopausal women**					**Postmenopausal women**				
	**Unweighted, n**		**OR (95% CI)**			**Unweighted, n**		**OR (95% CI)**		
	**Total**	**Event**	**Model 1**	**Model 2**	**Model 3**	**Total**	**Event**	**Model 1**	**Model 2**	**Model 3**
**Type 2 diabetes mellitus**										
**<12**	2335	64	0.825 (0.607, 1.121)	**1.467 (1.060, 2.030)**	0.976 (0.681, 1.400)	244	48	1.054 (0.725, 1.531)	1.371 (0.936, 2.008)	1.324 (0.917, 1.911)
**≥12**	10418	335	1 (Ref.)	1 (Ref.)	1 (Ref.)	12230	2374	1 (Ref.)	1 (Ref.)	1 (Ref.)
P value			*0.217*	***0.021***	*0.896*			*0.784*	*0.105*	*0.134*
**Hypercholesterolemia**										
**<12**	2335	174	**0.781 (0.641, 0.951)**	**1.305 (1.064, 1.600)**	1.083 (0.873, 1.344)	244	105	1.124 (0.827, 1.528)	1.241 (0.911, 1.690)	1.171 (0.864, 1.587)
**≥12**	10418	999	1 (Ref.)	1 (Ref.)	1 (Ref.)	12230	5153	1 (Ref.)	1 (Ref.)	1 (Ref.)
P value			***0.014***	***0.011***	*0.466*			*0.454*	*0.171*	*0.308*
**Hyper-LDL cholesterolemia**										
**<12**	2335	158	**0.807 (0.654, 0.995)**	**1.317 (1.056, 1.643)**	1.078 (0.851, 1.364)	244	104	1.183 (0.871, 1.607)	1.335 (0.980, 1.819)	1.260 (0.930, 1.708)
**≥12**	10418	892	1 (Ref.)	1 (Ref.)	1 (Ref.)	12230	5005	1 (Ref.)	1 (Ref.)	1 (Ref.)
P value			***0.045***	***0.015***	*0.534*			*0.283*	*0.067*	*0.136*

Data are odds ratios (95% CI); reference = ≥12 years. Total = unweighted participants per subgroup; Event = number with the disease. Bold indicates the 95% CI excludes 1. P-values are for the <12-vs-≥12 contrast.

Model 1: unadjusted. Model 2: adjusted for age. Model 3: adjusted for age, BMI, education (<13 vs. ≥13 yr), low income, ever smoking, heavy drinking, aerobic physical activity, hypertension; cholesterol outcomes additionally adjusted for diabetes, and diabetes for hypercholesterolemia.

Supplementary Table S2. Cardiometabolic disease control by age at menarche under the dichotomous classification (<12 vs. ≥12 years), with unweighted subgroup sizes and event counts, stratified by menopausal status.

Control	Premenopausal women					Postmenopausal women				
	Unweighted, n		OR (95% CI)			Unweighted, n		OR (95% CI)		
	Total	Event	Model 1	Model 2	Model 3	Total	Event	Model 1	Model 2	Model 3
**Type 2 diabetes mellitus**										
**<12**	64	17	0.799 (0.418, 1.529)	0.662 (0.331, 1.322)	0.738 (0.344, 1.587)	48	12	0.821 (0.395, 1.706)	0.849 (0.408, 1.765)	0.796 (0.386, 1.644)
**≥12**	335	86	1 (Ref.)	1 (Ref.)	1 (Ref.)	2374	645	1 (Ref.)	1 (Ref.)	1 (Ref.)
P value			*0.497*	*0.241*	*0.436*			*0.597*	*0.661*	*0.537*
**Hypercholesterolemia**										
**<12**	174	32	0.864 (0.548, 1.363)	**1.660 (1.006, 2.738)**	1.288 (0.735, 2.259)	105	55	0.727 (0.472, 1.120)	0.960 (0.618, 1.493)	0.878 (0.554, 1.391)
**≥12**	999	219	1 (Ref.)	1 (Ref.)	1 (Ref.)	5153	3053	1 (Ref.)	1 (Ref.)	1 (Ref.)
P value			*0.530*	***0.047***	*0.376*			*0.148*	*0.857*	*0.579*
**Hyper-LDL cholesterolemia**										
**<12**	158	36	0.945 (0.611, 1.461)	**1.859 (1.153, 2.997)**	1.591 (0.935, 2.708)	104	60	**0.634 (0.409, 0.984)**	0.840 (0.541, 1.303)	0.744 (0.471, 1.174)
**≥12**	892	232	1 (Ref.)	1 (Ref.)	1 (Ref.)	5005	3271	1 (Ref.)	1 (Ref.)	1 (Ref.)
P value			*0.798*	***0.011***	*0.087*			***0.042***	*0.436*	*0.204*

Data are odds ratios (95% CI); reference = ≥12 years. Total = unweighted participants per subgroup; Event = number with adequate control. Bold indicates the 95% CI excludes 1. P-values are for the <12-vs-≥12 contrast.

Model 1: unadjusted. Model 2: adjusted for age. Model 3: adjusted for age, BMI, education (<13 vs. ≥13 yr), low income, ever smoking, heavy drinking, aerobic physical activity, hypertension; cholesterol outcomes additionally adjusted for diabetes, and diabetes for hypercholesterolemia.
